# Integrins modulate the infection efficiency of West Nile virus into cells

**DOI:** 10.1099/vir.0.052613-0

**Published:** 2013-08

**Authors:** Katja Schmidt, Markus Keller, Bernhard L. Bader, Tomáš Korytář, Stefan Finke, Ute Ziegler, Martin H. Groschup

**Affiliations:** 1 Institute of Novel and Emerging Infectious Diseases, Friedrich-Loeffler-Institut, Federal Research Institute for Animal Health, Südufer 10, 17493 Greifswald – Isle of Riems, Germany; 2 Institute of Immunology, Friedrich-Loeffler-Institut, Federal Research Institute for Animal Health, Südufer 10, 17493 Greifswald – Isle of Riems, Germany; 3 Institute of Molecular Biology, Friedrich-Loeffler-Institut, Federal Research Institute for Animal Health, Südufer 10, 17493 Greifswald – Isle of Riems, Germany; 4 Nutritional Medicine Unit, Centre for Nutrition and Food Sciences, Technical University Munich, Gregor-Mendel-Straße 2, 85354 Freising, Germany

## Abstract

The underlying mechanisms allowing *West Nile virus* (WNV) to replicate in a large variety of different arthropod, bird and mammal species are largely unknown but are believed to rely on highly conserved proteins relevant for viral entry and replication. Consistent with this, the integrin αvβ3 has been proposed lately to function as the cellular receptor for WNV. More recently published data, however, are not in line with this concept. Integrins are highly conserved among diverse taxa and are expressed by almost every cell type at high numbers. Our study was designed to clarify the involvement of integrins in WNV infection of cells. A cell culture model, based on wild-type and specific integrin knockout cell lines lacking the integrin subunits αv, β1 or β3, was used to investigate the susceptibility to WNV, and to evaluate binding and replication efficiencies of four distinct strains (New York 1999, Uganda 1937, Sarafend and Dakar). Though all cell lines were permissive, clear differences in replication efficiencies were observed. Rescue of the β3-integrin subunit resulted in enhanced WNV yields of up to 90 %, regardless of the virus strain used. Similar results were obtained for β1-expressing and non-expressing cells. Binding, however, was not affected by the expression of the integrins in question, and integrin blocking antibodies failed to have any effect. We conclude that integrins are involved in WNV infection but not at the level of binding to target cells.

## Introduction

*West Nile virus* (WNV) is a small, enveloped, single-stranded RNA virus that belongs to the family *Flaviviridae* and to the genus *Flavivirus*. In the natural transmission cycle WNV circulates between mosquitoes as vectors and birds as reservoir hosts (Hurlbut *et al*., 1956; Work *et al.*, 1955). Most noticeably, WNV can infect a wide taxonomical range of vertebrate species but most of them do not sufficiently support virus replication for transmission (Dietrich *et al.*, 2005; van der Meulen *et al.*, 2005). Disease symptoms rarely occur, except in humans and horses where WNV infections are frequently accompanied by a mild fever (West Nile fever), which occasionally results in the development of neurological disorders with fatal outcome (Mostashari *et al.*, 2001; Murgue *et al.*, 2001; Petersen & Roehrig, 2001).

Determination of the factors that define host species tropism and pathogenesis includes the identification of receptors used by the virus for attachment to the cell surface and subsequent internalization, and whether this receptor is expressed at what density on the cell surface in a particular host or the respective organ or cell type (Marsh & Helenius, 1989; Schneider-Schaulies, 2000). Although a variety of putative *Flavivirus* receptors have been proposed to date, the array of cellular molecules required for virus entry has not been completely identified. Within the *Flavivirus* genus the early events in virus entry are best studied in *Dengue virus* and *Japanese encephalitis virus*.

The cellular receptors and determinants that mediate entry of WNV are unclear to date. The ability of WNV to infect a broad range of species (mosquitoes, reptiles, birds and mammals) and virtually every *in vitro* cell line is notable, and is supposed to be related to cellular proteins relevant for virus entry and replication that are highly conserved among divergent host species (Brinton, 2001, 2002).

The heterogeneous, ubiquitously distributed cell surface receptor integrin αvβ3 was described by Chu & Ng (2004) as the functional receptor for WNV, mediating both binding and entry. However, experiments accomplished by Medigeshi *et al.* (2008) resulted in a contrary conclusion. They demonstrated that WNV entry is independent of integrin αvβ3, since β3-integrin deficient cells could be infected successfully.

Integrins are highly conserved heterodimeric transmembrane proteins that mediate adhesion to the extracellular matrix and cell-to-cell contact, and which participate in many cell cycle processes (Clark & Brugge, 1995; Giancotti & Ruoslahti, 1999; Hynes, 2002). They consist of two non-covalently bound α and β glycoprotein subunits. In mammals, the combination of at least 18 α and eight β subunits yields 24 distinct integrin dimers that are expressed in large numbers on virtually all cell types (Gahmberg *et al.*, 2009; Hynes, 2002). Integrins are used as receptors by several viruses, including *Adenovirus* (Li *et al.*, 2001; Wickham *et al.*, 1993), *Foot-and-mouth disease virus* (Neff *et al.*, 1998), *Hantavirus* (Gavrilovskaya *et al.*, 1998, 1999), human coxsackievirus A9 (Roivainen *et al.*, 1994) and human echovirus 9 (Nelsen-Salz *et al.*, 1999).

The overall aim of this study was to clarify the involvement of integrins in WNV infection of cells. For this purpose we have established specific integrin knockout cell lines which lack the particular integrin subunits. Results of our study clearly demonstrate that the presence of αv-, β1- or β3-integrins is not required for the attachment of four different WNV strains to the cell surface. However, β1- and β3-integrin expression significantly enhances virus amplification. These findings imply that other routes are used in the absence of these integrins, or that different routes are generally used in parallel.

## Results

### Establishment of a cell culture model for infection experiments

Wild-type and specifically αv-deficient and β3-deficient cells were isolated from transgenic mouse embryos with specific modifications in the particular integrin subunit genomic sequences. The αv- and β3-knockout mice had been generated to investigate integrin-associated human diseases (Bader *et al.*, 1998; Hodivala-Dilke *et al.*, 1999; Hynes & Hodivala-Dilke, 1999; Reynolds *et al.*, 2002). In this study, a new αv-deficient mouse allele was applied. The αv-integrin subunit coding gene was inactivated by replacing the first exon with the *E. coli* LacZ gene cassette by homologous recombination (Fig. S1, available in *JGV* Online). With regard to the β3-integrin subunit, homologous recombination replaced a 1.4 kb fragment of the wild-type allele containing exon I and II by the 1.7 kb neomycin resistance cassette (Hodivala-Dilke *et al.*, 1999). Mouse embryonic fibroblasts (MEFs) were prepared from 12.5 day old mouse embryos. The β3-knockout mice are fully viable so that homozygous animals could be mated. In contrast, a homozygous αv knockout results in embryonic or perinatal death (Bader *et al.*, 1998). Therefore, heterozygous mice were mated to generate embryos with three different genotypes: αv (+/+) wild-type, heterozygous αv (+/LacZ) and homozygous αv-deficient (LacZ/LacZ) embryos. Embryos used for producing full αv-knockout cells exhibited the typical phenotype with intracerebral haemorrhages resulting from neurovascular defects as described previously (Fig. S2) (Bader *et al.*, 1998; McCarty *et al.*, 2002). The generated cell lines were designated wild-type MEFs, integrin αv-deficient MEFs or MEF-ITGAV(−/−) and integrin β3-deficient MEFs or MEF-ITGB3(−/−).

### Integrin expression properties of the mouse fibroblast cell lines

Cell lines were characterized with respect to their integrin expression features. Integrins are expressed as heterodimers on the cell surface. Thus, the loss of the αv-integrin subunit results in the lack of all five αv-integrin receptor combinations (αvβ1, αvβ3, αvβ5, αvβ6, αvβ8). In β3-deficient MEFs, only the expression of integrin αvβ3 is affected, since αIIbβ3 is exclusively expressed on thrombocytes. Two additional mouse fibroblast cell lines were included in the studies: mouse kidney fibroblasts MKF-ITGB1(−/−) and their parental cell line MKF-ITGB1(flox/flox), used as a wild-type control. Both cell lines differed from the MEFs described above in their morphology and in the proliferation rate. The absence of the integrin subunit β1 makes the expression of 12 heterodimeric integrin combinations, i.e. integrin αvβ1 and integrins α(1–11)β1, impossible.

**Fig. 1.**
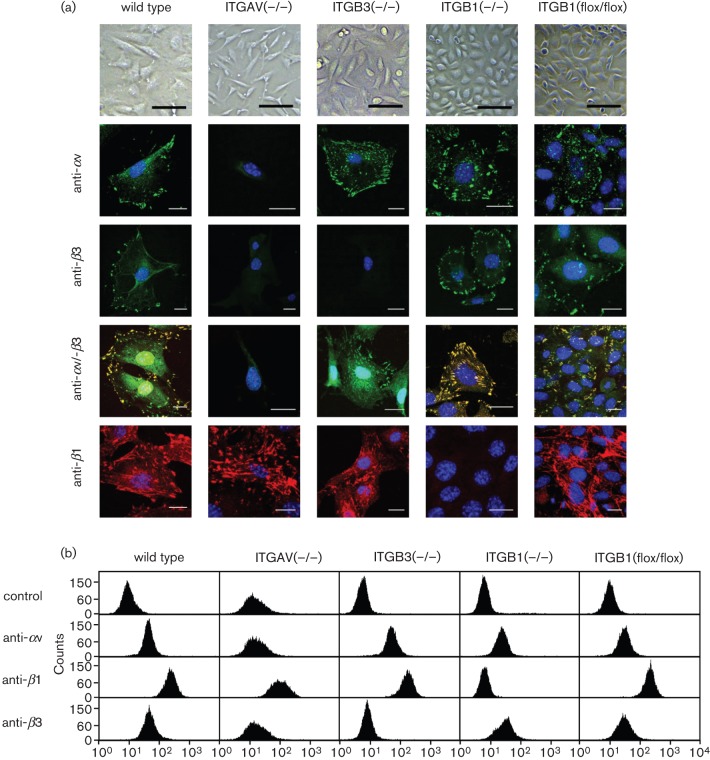
Integrin expression of mouse fibroblast cell lines. (a) Confocal microscopy images of integrin expression and distribution as RGD overlays for each combination cell line×antibody. Antibodies specific for the integrin subunits αv, β1 or β3 and the combination of αv-integrin specific (green) and β3-integrin specific (red) antibodies were used for staining. Merged colours of double staining indicate co-localization of integrins (yellow). Scale bars of 100 µm (light microscopy) and 20 µm (fluorescence images) are displayed. (b) Fluorescence profiles from flow cytometry analysis. Cells were incubated with integrin specific antibodies or left untreated (cell controls; isotype controls not shown here). Panels represent FACS histogram for each combination of cell line×antibody. Abscissa, fluorescence intensity (log scale); ordinate, number of cells.

In order to verify the αv-, β1- and β3-integrin deficiency of these cell lines, indirect fluorescent antibody (IFA) immunofluorescence measurements and flow cytometry were accomplished. Confocal laser scanning microscopy allowed visualizing the integrin distribution in focal contact points on the cell surface of the mouse fibroblasts, in particular around the cell protrusions and cell-to-cell-contacts. As seen in Fig. 1(a) imaging showed that αv- and β3-integrins assembled in a typical focal contact manner, i.e. isolated bar-shaped dots localized on the cell surface, whereas β1-integrins presented a more elongated assembly similar to those of stress fibres with which they are connected (Kusano *et al.*, 2000; Liu *et al.*, 2010).

Flow cytometry was carried out to estimate integrin expression levels (Fig. 1b). Results were generally consistent with the PCR data (not shown) and with IFA results, and verified the absence of αv- and β3-integrins on MEF-ITGAV(−/−), of β1-integrins on MKF-ITGB1(−/−) and of β3-integrins on MEF-ITGB3(−/−) cells. Interestingly, expression levels of individual integrins were not affected by the integrin subunit knockdowns, e.g. β1-integrin was not upregulated as compensation in β3-integrin deficient cells, and vice versa, which is consistent with what has been previously reported (Bader *et al.*, 1998; Hodivala-Dilke *et al.*, 1999).

### Integrin deficient cells are not resistant to WNV infection

**Fig. 2.**
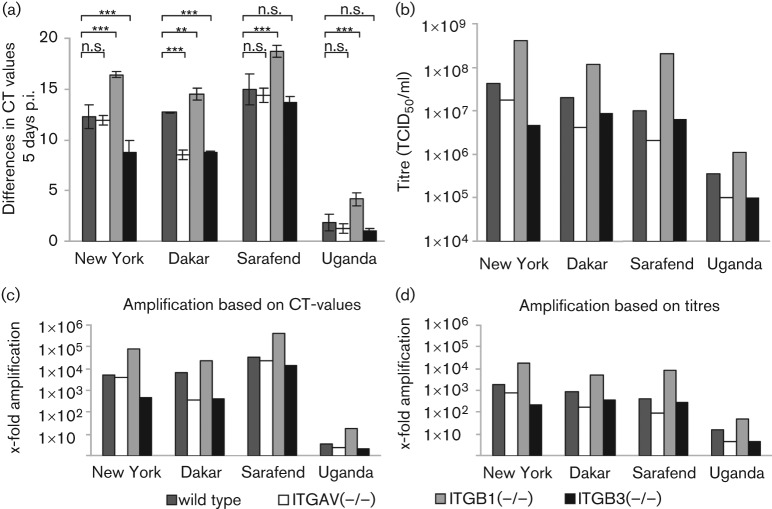
Permissiveness of integrin deficient mouse fibroblasts for WNV. (a) CT values in the supernatant 5 days post infection (p.i.) were subtracted from those at the onset of infection to estimate the increase in genome containing particles of each combination cell line×virus strain. (b) Representative samples, based on CT values, were titrated to check if infectious virus yields were comparable to the amount of viral RNA. (c, d) To facilitate the presentation of data, the increase in original viral genome equivalents after 5 days of incubation was expressed in x-fold amplification as a multiple of viral particle numbers based on CT values (c), calculated from the CT value differences shown in (a), and TCID_50_ titres determined on Vero cells (d), calculated from titres shown in (b), in relation to CT values or titres, respectively, at the time of infection is shown. Wilcoxon–Mann–Whitney test (two-sided testing); multiple testing, with Bonferroni correction: n.s., *P*>0.05; **, 0.001<*P*≤0.01; ***, *P*≤0.001. Error bars represent±1 sd (*n* = 3 independent assays with triplicates each).

In a first experiment the ability of WNV to infect and to replicate in cells lacking the αv- or β3-integrin subunit was assessed in a qualitative approach. The experiment was designed to confirm results from Chu & Ng (2004) and to study whether there are strain dependent differences in the infection ability. Wild-type MEFs were used as a control cell line. Since β1-integrins had not been found to affect WNV infectivity greatly, β1-deficient cells were also included in the experiment as a control. Cells were infected with four WNV strains: two WNV lineage 1 strains (New York 1999, Dakar) and two lineage 2 strains (Uganda 1937, Sarafend) and incubated for 5 days (time period usually used for propagation). A low m.o.i. was chosen to allow long-term virus propagation. Relative amounts of viral RNA content in the supernatants were quantified by qRT-PCR (Fig. 2a). To calculate the yields of infectious virus particles, the titres of representative samples, based on cycle threshold (CT) values, were determined by TCID_50_ assay (Fig. 2b). Surprisingly, results clearly show that all cell lines were permissive for WNV and that all strains used in this study were able to replicate in the above-mentioned cell lines. Obviously, replication of WNV could not be prevented completely by the absence of one or several integrins. There were, however, distinct and reproducible differences in the CT values measured after 5 days. Significantly reduced virus yields were found in the supernatant of β3-deficient MEFs when infected with the WNV strains New York and Dakar relative to wild-type MEFs. Amplification of WNV Dakar in αv-deficient cells was also significantly lower. The highest replication efficiencies were seen in β1-deficient cells which were comparable to those found in Vero cells (data not shown). The significantly enhanced virus propagation in comparison to wild-type cells can presumably be attributed to the fact that MKF-ITGB1(−/−) were derived from a different tissue and had been immortalized. Titration results confirmed these findings, although amplification rates of infectious virus particles in terms of TCID_50_ titres were lower than the amplification of viral genome equivalents (Fig. 2c, d).

### Rescue of β3-integrins in β3-deficient cells

**Fig. 3.**
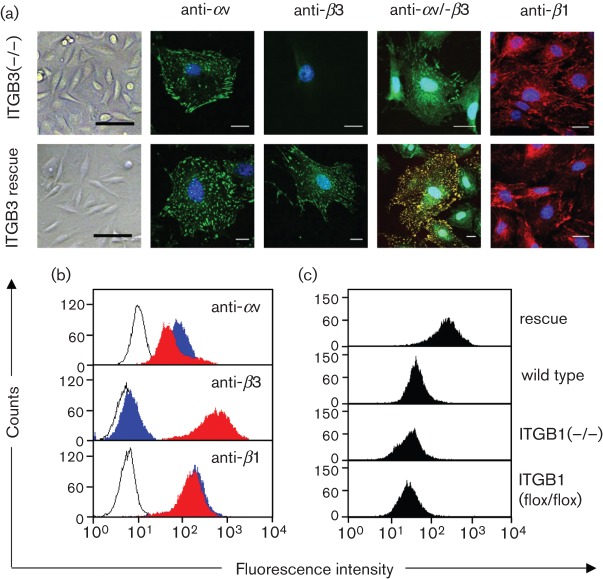
Integrin expression of integrin β3-rescue MEFs. (a) Immunofluorescence images of β3-deficient MEFs (top row) and β3-rescue MEFs (bottom row). Anti-αv-, anti-β3- and anti-β1-antibodies were used for the staining. Double fluorescence staining depicts co-localization (yellow) of αv- (green) and β3- (red) integrins. Scale bars of 100 µm (light microscopy) and 20 µm (fluorescence images) are displayed. (b) Expression profiles of β3-rescue MEFs. FACS histograms illustrate the fluorescence intensities of the different antibodies applied on β3-deficient (blue profiles) and β3-rescue MEFs (red profiles). Fluorescence intensities of unstained cell controls are displayed as open profiles. (c) The β3-integrin levels of the β3-rescue MEFs were compared to those of wild-type MEFs, β1-deficient MKFs and β1-floxed MKFs in terms of fluorescence intensities when those of the cell controls were set to the same level (mean fluorescence intensity of 10). Abscissa, fluorescence intensity (log scale); ordinate, number of cells.

Although our findings had shown that αv-, β1- and β3-integrins were not essential for a productive WNV infection, it became evident that the replication in β3-integrin defective cells was significantly reduced. To unambiguously confirm this finding, and to exclude possible side effects the mouse line background may have on derived mouse fibroblast, a functional rescue was accomplished by transfecting MEF-ITGB3(−/−) cells with an expression plasmid encoding the full mouse integrin β3-subunit (designated β3-rescue MEFs or MEF-ITGB3(−/−) rescue) followed by zeocin selection for stably transfected cells. Transfected cells were analysed by IFA (Fig. 3a), and flow cytometry was used to enrich β3-integrin expressing cells by sorting (final purity 99 %). MEF-ITGB3(−/−) cells strongly expressed β3-integrins without affecting the αv- and β1-integrin cell surface expression and distribution patterns. FACS analysis gave similar qualitative results (Fig. 3b) and revealed that β3-rescue MEFs considerably overexpressed β3-integrins on their surfaces as compared to other integrin β3-expressing mouse fibroblasts (Fig. 3c).

### Independence of WNV binding from β1- and β3-integrin expression

**Fig. 4.**
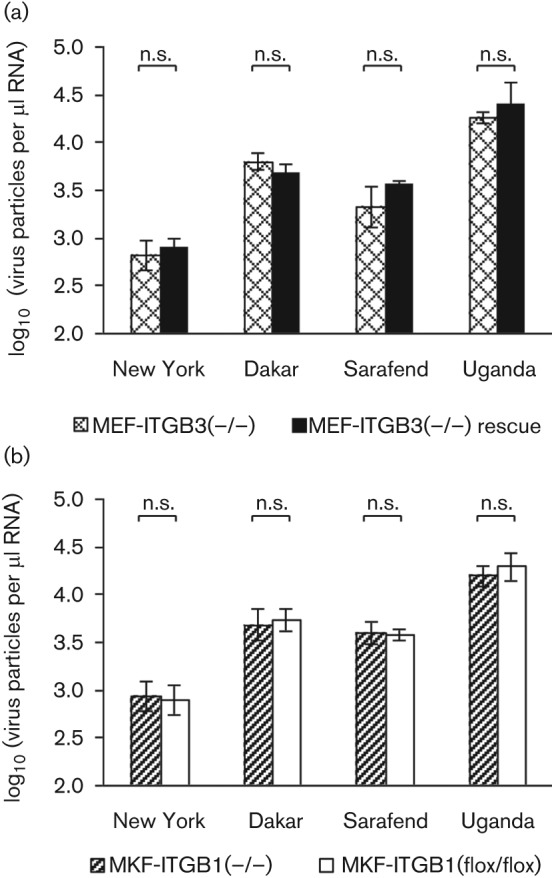
WNV binding to specifically integrin deficient and integrin expressing cells. (a) WNV binding to integrin β3-deficient and β3-rescue MEF. (b) WNV binding to integrin β1-deficient and to the corresponding integrin expressing MKFs. Viral particle numbers, adsorbed to the cell monolayer, were calculated from CT values determined by qRT-PCR. Yields of four different WNV strains are shown by the amount of virus particles per µl RNA (log-transformed). Error bars represent ±1 sd of transformed means (*n* = 3 independent assays each in triplicate). n.s., *P*>0.05 (two-way ANOVA).

β1- and β3-deficient mouse fibroblasts were infected in parallel with the corresponding integrin β1- or β3-expressing cell line to facilitate comparisons in order to allow direct quantitative assessment. After pre-incubation at 4 °C to synchronize infection and to prevent virus internalization, cells were subsequently infected with the four WNV strains. After virus adsorption for 1 h, unbound virus was removed by extensive washing before viral RNA yields of cell-adsorbed virus particles were assessed by qRT-PCR (Fig. 4).

Viral yields bound to integrin β3-expressing MEF-ITGB3(−/−) rescue and non-expressing MEF-ITGB3(−/−) cells did not differ significantly (*P*>0.05, two-way ANOVA) (Fig. 4a). The virus strain, however, had a significant effect on the virus particle numbers. This fact was attributed to the different amounts of original viral genome containing particle numbers since the calculation of the m.o.i. was based on the determination of infectious virus particles by plaque assay. This led to high systematic differences in the original viral genome containing particle numbers among the four WNV strains (New York < Sarafend < Dakar < Uganda).

Similar experiments were conducted with the focus on β1-integrins. Since the comparison of immortalized β1-deficient MKFs with the generated wild-type MEFs was not meaningful, MKF-ITGB1(flox/flox) were used as a wild-type control to investigate the role of β1-integrins on WNV infection. Effectively, no significant difference in binding to the integrin expressing MKF-ITGB1(flox/flox) and non-expressing MKF-ITGB1(−/−) cell lines was found for any of the four WNV strains (Fig. 4b).

### Enhanced replication of WNV in integrin β1- and β3-expressing cells

**Fig. 5.**
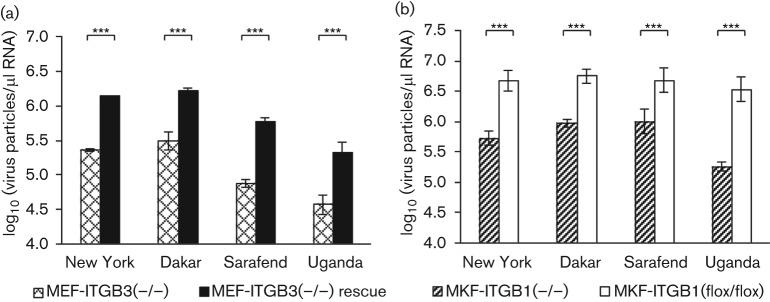
Replication of four WNV strains in integrin expressing and non-expressing cells. (a) WNV replication in integrin β3-deficient and β3-rescue MEFs. (b) Replication in integrin β1-deficient and β1-expressing cells. Viral particle numbers in the supernatant 48 h post-infection were calculated from CT values determined by qRT-PCR. Concentrations expressed as virus particles per µl RNA were log-transformed. Error bars represent±1 sd of transformed means (*n* = 3 independent assays each in triplicate). ***, *P*≤0.001 (two-way ANOVA, multiple comparisons Bonferroni corrected).

In a parallel approach to the binding experiment, another set of cells was shifted to 37 °C after cold infection to measure the internalization of cell-bound virus particles and to investigate the virus replication capacities of the particular integrin deficient and expressing mouse fibroblasts. Forty eight hours after infection an obvious cytopathic effect was seen in the β3-rescue MEFs, whereas β3-deficient MEFs seemed less affected, when compared to mock infected control cells. Clear differences in the amount of viral RNA two days after infection were observed. The level of viral genome containing particles in the supernatant of the β3-expressing rescue cells was always higher than in the β3-deficient cells (Fig. 5a). The cell line and the virus strain were found to have a highly significant effect on viral yields. The effect of the factor virus strain, however, is attributed to differences in the original viral genome numbers (see above) and is therefore irrelevant. The statistical interaction cell line×virus strain was not significant (*P*>0.05, Scheirer–Ray–Hare test). This means that the differences in viral replication between deficient and rescue cells were essentially the same for all strains. The highly significant effect of the factor cell line indicates that β3-integrins considerably facilitate virus replication. The relative virus yields in the supernatant of MEF-ITGB3(−/−) were less than 20 % of yields in integrin β3-rescue MEFs in most cases.

Similar findings were observed in the ITGB1 replication experiment. Replication within 48 h after infection was considerably higher in MKF-ITGB1(flox/flox), used as a wild-type control, regardless of the virus strain used (Fig. 5b). The factor cell line had a highly significant effect on the virus yield, indicating that the amount of virus produced was significantly increased in the presence of β1-integrins. The statistical interaction of cell line×virus strain also showed no significance (*P*>0.05, Scheirer–Ray–Hare test). The lack of β1-integrins resulted in a reduction of produced virus particles of more than 70 % up to 90 %.

### Integrin blocking antibodies have no effect on WNV binding or internalization

A competition assay was performed using integrin specific blocking antibodies to verify that β1- and β3-integrins do not play a role in WNV binding. β1-floxed MKFs and β3-rescue MEFs were incubated with increasing concentrations of blocking antibodies (0, 5, 15, 25, 50 µg ml^−1^) targeting the β1- (HMβ1-1) and the β3-subunit (HM beta 3.1), both of which block ligand binding of integrins in functional assays (Piali *et al.*, 1995; Ridger *et al.*, 2001; Sangaletti *et al.*, 2008). As shown earlier in this study, both antibodies recognized the corresponding integrin subunit of the mouse fibroblasts. Cells were incubated with either one or both antibodies (5; 25 µg ml^−1^). Binding of antibodies was allowed for 1 h at 4 °C before cells were washed to remove unbound antibodies and infected at an m.o.i. of 4 p.f.u. per cell (β1-floxed MKFs) or 20 p.f.u. per cell (β3-rescue MEFs) before incubation proceeded at 4 °C (binding assay) or 37 °C (replication assay). In accordance with our previous findings the application of integrin blocking antibodies did not affect virus binding to cells and the internalization in terms of virus production within 48 h (Fig. S3). No correlations were seen between antibody concentration and the number of bound or produced virus particles (coefficient of determination (*r*^2^) below 0.08 for the binding set, except for double application on β3-rescue MEFs which yielded 0.383, and below 0.13 for the replication set). In addition, the simultaneous application of both antibodies had no effect.

## Discussion

The current understanding of WNV binding to and entry into host cells is rather incomplete. Previous studies using host gene knockdown assays as well as specific antibody binding competition assays suggested that integrins, in particular integrin αvβ3, act as the functional receptors for this virus (Chu & Ng, 2004). In line with this, CS-1 melanoma cells, highly differentiated non-invasive tumour cells that do not synthesize β3- and β5-integrins (Knudsen *et al.*, 1982), were non-permissive to WNV (Chu & Ng, 2004). However, contradicting results were found by Medigeshi *et al.* (2008), who were able to infect CS-1 melanoma cells with WNV with efficiencies comparable to those in other cell lines. It is known that integrin expression on CS-1 melanoma cells can be induced by certain stimuli (Thomas *et al.*, 1993).

In order to resolve the inconsistent results on the involvement of integrin in WNV infection of cells we have established distinct specific integrin subunit deficient cell lines. The integrin knockout and rescued mouse fibroblast cell lines appear to provide a more reliable model than CS-1 melanoma cells. The ablation of one integrin subunit leads to the absence of all corresponding integrin heterodimer combinations on the cell surface. Integrin heterodimers, which fulfil important cellular functions, are usually encoded redundantly by more than one subunit combination. Whether the loss of either the αv-, β1- or β3-subunit modulated the expression levels of other integrin heterodimer combinations was beyond the scope of this study. The cell culture model was designed: (i) to study the involvement of integrins and the extent to which they participate in WNV replication, (ii) to identify which subunit is involved, (iii) to clarify their function in WNV binding, and (iv) to investigate whether or not specific traits of lineages or even strains determine the affinity to these surface molecules.

In this study, there was no evidence that WNV binding to mouse fibroblasts was in any way influenced by the expression of the integrins when integrin expressing and non-expressing cells were compared as to their binding efficiencies. Equally, application of integrin specific antibodies that block integrin binding to their ligands failed to inhibit virus binding and infection of cells, even at high concentrations. This is in contrast to the results of Chu & Ng (2004), who inhibited to a large extent, though not completely, both binding and internalization of WNV by application of function blocking antibodies. It can be argued that the different outcome of our study may result from the use of different cell lines and antibodies. Thus, the overriding importance of integrin αvβ3, at least its role as a binding receptor, must be denied.

Our results clearly show that the integrins in question are not a prerequisite for an infection of cells by WNV. To enhance the comparability of results on how β1- and β3-integrin expression affects WNV infectivity, and to facilitate a direct quantitative assessment, deficient cell lines were directly compared with their corresponding integrin expressing cell lines. This allowed any background differences between cell lines that may arise from possible differences between mouse strains to be excluded. Although we were not able to confirm that integrins participate in virus adsorption to cells, it could be demonstrated in this study that integrins enhance replication of WNV in the mouse fibroblast cells. The replication efficiency of WNV was clearly reduced by 90 % (at maximum) by the lack of either the β1- or β3-integrin subunit. These results were generally in accordance with those obtained by Chu & Ng (2004), showing the loss of the β3-integrin subunit had the greatest effect on cell permissiveness in terms of viral yields. Since the expression of both β-integrins seems to affect virus replication it is likely that several integrin receptors may be used in parallel by WNV and that in the absence of specific integrins the virus can use other members of the integrin family.

Our experimental approach for the infection assays was not able to distinguish whether integrins are required for entry or for another stage of the WNV replication cycle. An increasing number of viruses have implicated integrins with different functions beyond virus binding, e.g. entry by initiating endocytosis, rearrangement and modification of the cytoskeleton, activation of signalling pathways or by providing a more stable environment for the virus by downregulating the innate immune response mechanisms of the host cell (Gao *et al.*, 2008). For example, echovirus 1 and coxsackievirus A9 enter cells via integrin-mediated endocytosis regulated by a dynamin-dependent mechanism (Heikkilä *et al.*, 2010; Pietiäinen *et al.*, 2004), *Dengue fever virus* binding to the integrin αvβ3 induces actin cytoskeleton rearrangement (Zhang *et al.*, 2007), and Herpes simplex virus is routed to lipid rafts and dynamin-2-dependent acidic compartments upon binding integrin αvβ3 (Gianni *et al.*, 2010b). However, there is currently no evidence that HSV directly interacts with integrin αvβ3 (Gianni *et al.*, 2010a).

Integrins are not only the main activators in linking extracellular stimuli to the cytoskeleton, inducing actin rearrangement, and in regulating the cell cycle (Clark & Brugge, 1995; Giancotti & Ruoslahti, 1999; Yamada & Miyamoto, 1995). Remarkable in terms of viral entry is their ability to guide the trafficking of other signalling receptors, in particular of growth factor receptors, as well as cellular structures such as cholesterol-rich membrane microdomains, and they influence the way by which growth factor receptors respond to their ligands (Caswell *et al.*, 2009; Walker *et al.*, 2005). Integrins are concentrated in lipid rafts which are docking sites for many signalling molecules. Hence they may also function as modulators of the intensity of multiple other signalling cascades (Baron *et al.*, 2003; Giancotti & Tarone, 2003; Krauss & Altevogt, 1999).

Our findings suggest specific functions of integrins in WNV infection: (i) integrins may interact as a co-receptor directly with the virus in combination with another surface receptor, or (ii) they may not directly interact with the virus but enhance or block other cell surface molecules important for WNV binding and entry, or may improve accessibility to other surface receptors for virus binding, or (iii) integrins may merely be involved in the downstream signal transduction, which either initiates viral uptake or modifies the cellular milieu for virus replication in a post-entry step. Though an involvement of integrins in another stage of the virus replication cycle cannot be excluded, the surface localization of these cell adhesion molecules leads to the assumption that integrins respond to virus binding, and may therefore be involved in virus internalization rather than in the downstream replication cycle (Gao *et al.*, 2008; Triantafilou *et al.*, 2001).

Since disagreement in the essentials of the studies by Chu & Ng (2004) and Medigeshi *et al.* (2008) in respect of the involvement of integrin αvβ3 in WNV entry was thought to result from the genetic differences between WNV strains, four representative strains of the two major WNV lineages were selected for this present study, including both of those used in these two studies. Observations made from infection experiments with flaviviruses suggest that receptor binding and entry characteristics may depend on the serotype or strain used (Bielefeldt-Ohmann *et al.*, 2001; Liu *et al.*, 2004). However, our data do not adumbrate strain dependent effects in WNV regarding the usage of integrins during attachment, entry or the replication process.

Altogether, our data indicate that integrins play a significant role in WNV infection of cells. Still, the understanding of the WNV–integrin relationship is superficial and needs further investigation in order to convincingly depict the complex interaction. The specification of the molecules involved and the entry-associated signalling processes that finally lead to virus internalization will definitely help to elucidate the first steps in WNV infection, and to identify the factors that modulate the susceptibility to and the transmission efficiency of WNV.

## Methods

### 

#### Cell lines.

Vero 76 cells, Vero B4 cells and culture media were obtained from the Collection of Cell Lines in Veterinary Medicine, Isle of Riems, Germany. Vero cells were cultured in ZB5 containing 10 % FBS and grown under standard conditions. The integrin β1-deficient mouse kidney fibroblasts MKF-ITGB1(−/−) and their parental cell line MKF-ITGB1(flox/flox) were kindly gifted by R. Faessler. Mouse fibroblasts were grown in high glucose medium, ZB 10 supplemented with 10 % FBS.

#### Viruses.

The WNV New York 1999 flamingo isolate (GenBank accession no. AF196835) and WNV Uganda 1937 (accession no. M12294) were kindly provided by Matthias Niedrig. WNV Sarafend (accession no. AY688948) was a generous gift from Arno Mullbacher. WNV Dakar was purchased from the National Collection of Pathogenic Viruses, UK. The identity of the virus strains was confirmed by sequencing the viral envelope protein encoding sequence. Vero 76 cells were used throughout this study to propagate the WNV strains. Virus containing culture supernatants were cleared by low speed centrifugation and aliquots were stored at −70 °C for further use.

#### Antibodies.

Hamster anti-mouse CD61 (HM beta 3.1), without labelling, or labelled with biotin or Alexa Fluor 488, directed against the mouse integrin β3, and the rat anti-mouse CD51 (RMV-7), unlabelled or Alexa Fluor 488 labelled, recognizing the mouse integrin αv-subunit were purchased from AbD Serotec. The FITC-labelled mouse integrin β1-specific antibody HMβ1-1 and the Armenian hamster IgG controls were purchased from Santa Cruz Biotechnology. The LEAF purified antibodies for function blocking assays against the β1-(HMβ1-1) and β3-(HM beta 3.1) integrin subunits, as well as the Alexa Fluor 647 labelled antibody clones (HM beta 3.1, HMβ1-1) and the LEAF purified Armenian hamster IgG isotype were purchased from BioLegend. The secondary antibodies goat anti-rat IgG Alexa Fluor 488 and goat anti-hamster IgG DyLight 488 were obtained from Invitrogen and AbD Serotec, respectively.

#### Viral infectivity.

Viral infectivity was determined on Vero cells following standard procedures (see supplementary material for details).

#### Preparation of mouse embryonic fibroblasts.

The β3-deficient mice were generously gifted by Kairbaan Hodivala-Dilke. αv-deficient mice were generated as described in the supplementary material. Mouse embryos were isolated at embryonic day 12.5 from the female uteri. MEFs were prepared as described elsewhere (Bader *et al.*, 1998) (see supplementary material for details).

#### Transfection of cells and cell separation.

The cDNA of the mouse β3-integrin subunit including the signal sequence was synthesized and cloned into the eukaryotic expression plasmid pcDNA 3.1/zeo(+) (Invitrogen) by use of its *Bam*HI and *Not*I restriction sites (GeneArt). The sequence based on the mRNA integrin β3-mouse sequence was provided by GenBank (accession no. NM016780.2). Transfection of MEFs was performed using 4 µg of the integrin β3-encoding plasmid mixed with the Lipofectamine 2000 transfection reagent (Invitrogen) at a ratio of 1 : 3 (µg µl^−1^) as specified by the manufacturer. Selection in order to raise stably transfected cells was implemented by addition of 2 mg zeocin ml^−1^ (Invitrogen) to the growth medium.

Transfected cells were separated to increase the percentage of positive, integrin expressing cells within the population. For positive selection the MACS system (Miltenyi Biotec) was used and the manufacturer‘s instructions were generally followed. Cells were detached and resuspended in sterile PBS containing 0.5 g sodium azide l^−1^ before being incubated with biotin-labelled integrin β3-antibody HM beta 3.1 for 2 h. After incubation with anti-biotin MicroBeads (Miltenyi Biotec), integrin β3-positive and -negative cells were separated using MS columns.

#### IFA and flow cytometry.

Cells were stained with integrin subunit specific antibodies to visualize integrin expression patterns by confocal laser scanning microscopy and to evaluate integrin expression levels by flow cytometry analysis (see supplementary material for details).

#### Long-term replication assay.

Vero cells and mouse fibroblasts prior to confluency (80–95 %) were infected with m.o.i. 0.05 p.f.u. per cell in a total volume of 1 ml virus diluted in medium without FBS. Virus absorption was allowed for 1 h at 37 °C with gentle agitation every 20 min. After addition of fresh maintenance medium, supplemented with 25 mM HEPES and 2 % FBS, incubation proceeded at 37 °C. Five days after infection, the supernatant was harvested and cleared from cell debris by low speed centrifugation.

#### Binding assay.

Cells were seeded on 12-well culture dishes before infection. In a first approach the exact seeding concentration of each cell line was determined to give same cell densities per well 24 h later when infected (1×10^5^ MEF-ITGB3(−/−) and MEF-ITGB3(−/−)rescue, or 5×10^5^ MKF-ITGB1(−/−) and MKF-ITGB1(flox/flox)). After pre-incubation at 4 °C cells were washed with FBS-free medium containing 0.05 g BSA fraction V l^−1^ before inoculation with virus at 10 p.f.u. per cell (ITGB3 experiments) or 2 p.f.u. per cell (ITGB1 experiments). Incubation was allowed for 1 h at 4 °C with intermittent agitation. To remove unbound virus particles the virus suspension was aspirated and cells were washed three times with PBS. Finally, cell monolayers were dissolved in TRIzol (Invitrogen). For the application of integrin blocking antibodies the experiments were instead carried out on 24-well culture dishes with half the cell number.

#### Replication assay.

Following the above described procedure, after binding at 4 °C for 1 h, plates were shifted to 37 °C and cells were incubated for 30 min to allow endocytosis. Cells were washed once with PBS before being treated with acid glycine buffer pH 3.0 for 1 min to inactivate non-internalized virus (Hung *et al.*, 1999), followed by washing twice with PBS. Finally, fresh maintenance medium containing 2 % FBS was added to the wells. The culture supernatant was harvested after incubation at 37 °C for 48 h.

#### RNA extraction and qRT-PCR.

Viral and total RNA extracts were obtained as described in the supplementary material. Five microlitres of the eluted viral RNA were used to determine viral genome containing particle numbers by quantitative reverse-transcriptase PCR as described by Eiden *et al.* (2010). For quantifying the WNV genome copy numbers through a calibration curve, serial dilutions of a synthetic RNA control were run in parallel. The qRT-PCR was run on the Mx 3000P QPCR (Stratagene) or CFX96 Real-Time Systems (Bio-Rad).

#### Statistical analysis.

Statistical significance values of results were determined using JMP version 3.2.1 (SAS Institute, USA). Data from infection experiments were log-transformed, if necessary, to stabilize variances with respect to parametric statistical testing. The results of all parametric ANOVA tests were checked with the non-parametric Scheirer–Ray–Hare test (Scheirer *et al.*, 1976). If the results of the parametric ANOVA were in accordance with the Scheirer–Ray–Hare test, linear contrasts were tested with the parametric ANOVA to identify significant differences of means among groups.
